# Participatory action research to identify a package of interventions to promote postpartum family planning in Burkina Faso and the Democratic Republic of Congo

**DOI:** 10.1186/s12905-018-0573-5

**Published:** 2018-07-05

**Authors:** Nguyen Toan Tran, Wambi Maurice E. Yameogo, Félicité Langwana, Mary Eluned Gaffield, Armando Seuc, Asa Cuzin-Kihl, Seni Kouanda, Désiré Mashinda, Blandine Thieba, Rachel Yodi, Jean Nyandwe Kyloka, Tieba Millogo, Abou Coulibaly, Basele Bolangala, Souleymane Zan, Brigitte Kini, Bibata Ouedraogo, Fifi Puludisi, Sihem Landoulsi, James Kiarie, Suzanne Reier

**Affiliations:** 1 Department of Reproductive Health Research, World Health Organization, Avenue Appia 20, 1211 Genève 27, Switzerland; 2 Institute of Demography and Socioeconomics (IDESO), University of Geneva, Boulevard du Pont d’Arve 40, 1211 Geneva, Switzerland; 3 Australian Centre for Public and Population Health Research, Faculty of Health, University of Technology, PO Box 123, Sydney, NSW 2007 Australia; 4 Institut de Recherche en Sciences de la Santé, 03 B.P. 7192, Ouagadougou 03, Burkina Faso; 5 Institut Africain de la Santé Publique, 12 B.P. 199, Ouagadougou, Burkina Faso; 6 School of Public Health, University of Kinshasa, Kinshasa, Democratic Republic of the Congo; 7 World Health Organization Country Office in Burkina Faso, 158 Av. de l’Indépendance, 03, Ouagadougou 03, BP 7019 Burkina Faso; 8 World Health Organization Country Office in the Democratic Republic of Congo (DRC), Avenue des Cliniques N°42, BP 1899 Kinshasa I, Democratic Republic of the Congo; 9 Centre Médical de Bokin, District sanitaire de Yako, Région du Nord, Burkina Faso; 10 Centre Mère et Enfant (CME) de Bumbu, avenue Mafuta 48/49, Commune de Bumbu, Province de Kinshasa Democratic Republic of the Congo

**Keywords:** Postpartum family planning, Pregnancy, Antenatal care, Postnatal care, Public health intervention package, Health service strengthening, Contraceptive uptake

## Abstract

**Background:**

The YAM DAABO study (“your choice” in Mooré) takes place in Burkina Faso and the Democratic Republic of Congo. It has the objective to identify a package of postpartum family planning (PPFP) interventions to strengthen primary healthcare services and determine its effectiveness on contraceptive uptake during the first year postpartum. This article presents the process of identifying the PPFP interventions and its detailed contents.

**Methods:**

Based on participatory action research principles, we adopted an inclusive process with two complementary approaches: a bottom-up formative approach and a circular reflective approach, both of which involved a wide range of stakeholders. For the bottom-up component, we worked in each country in three formative sites and used qualitative methods to identify barriers and catalysts to PPFP uptake. The results informed the package design which occurred during the circular reflective approach – a research workshop gathering service providers, members of both country research teams, and the WHO coordination team.

**Results:**

As barriers and catalysts were found to be similar in both countries and with the view to scaling up our strategy to other comparable settings, we identified a common package of six low-cost, low-technology, and easily-scalable interventions that addressed the main service delivery obstacles related to PPFP: (1) refresher training of service providers, (2) regularly scheduled and strengthened supportive supervision of service providers, (3) enhanced availability of services 7 days a week, (4) a counseling tool, (5) appointment cards for women, and (6) invitation letters for partners.

**Conclusions:**

Our research strategy assumes that postpartum contraceptive uptake can be increased by supporting providers, enhancing the availability of services, and engaging women and their partners. The package does not promote any modern contraceptive method over another but prioritizes the importance of women’s right to information and choice regarding postpartum fertility options. The effectiveness of the package will be studied in the experimental phase. If found to be effective, this intervention package may be relevant to and scalable in other parts of Burkina Faso and the DRC, and possibly other Sub-Saharan countries.

**Trial registration:**

Retrospectively registered in the Pan African Clinical Trials Registry (PACTR201609001784334, 27 September 2016).

**Electronic supplementary material:**

The online version of this article (10.1186/s12905-018-0573-5) contains supplementary material, which is available to authorized users.

## Background

By spacing the birth-to-pregnancy interval by at least 2 years and the inter-birth interval by at least 3 years, effective postpartum contraceptive use in less developed countries could help to prevent poor maternal, perinatal, and neonatal health outcomes, including stillbirth, prematurity, low birth weight, neonatal and maternal mortality [1–4]. Routine reproductive, maternal, newborn, and child health (RMNCH) services offer frequent points of contact between providers and women, which can be used as opportunities to address the contraceptive needs of postpartum women and couples [5]. Despite observed advances in access to RMNCH services in many sub-Saharan African countries in recent decades, progress is weak regarding effective postpartum contraceptive use [6–8]. The unmet need for postpartum contraception right after birth remains unacceptably high at 75% for the West and Central Africa region where our study takes place [9].

In 2013, the World Health Organization (WHO) and key partners published the programming strategies for postpartum family planning (PPFP) to support program managers in their efforts to integrate PPFP into national and local health strategies [10]. This document identifies convenient entry points for PPFP interventions into the RMNCH continuum: antenatal care (ANC); labor and delivery; postnatal care (PNC); immunization; and child health. The following interventions noted in the document may have a positive effect on postpartum contraceptive uptake: (1) counseling activities in ANC [11–15]; (2) provision of PPFP information, education, and counseling materials before the woman is discharged home from the health facility, including the provision of emergency contraception backup for lactational amenorrhea method (LAM) users [16–19]; (3) access to contraceptive methods (including intrauterine devices (IUD)) immediately after childbirth [20–22]; (4) provider competencies in quality counseling and the provision of quality services with a range of readily available products [23–25]; (5) longer programs with several contact points between providers and clients across the continuum of care versus short ANC interventions [26–28]. We need however more trials that explore the desires, intentions, and priorities of women or couples related to PPFP options and that integrate providers’ and other stakeholders’ views, all of which may differ across settings. The field also needs more trials that give a detailed description of the methods and tested interventions, which was not always the case in previous studies.

### Study objective, hypotheses, and frameworks

In light of the socio-cultural and health service challenges related to contraception in general and to PPFP in particular, we launched a multipronged and multifaceted operational research intervention (2015–2018): the YAM DAABO study (i.e., “your choice” in Mooré, a national language in Burkina Faso). The overall objective of the study is to determine the effectiveness of a contextually-sensitive, primary-healthcare-focused intervention package on the uptake of contraceptive methods during the first year postpartum.

Based on the different entry points into the RMNCH continuum and the published literature [26–28], we assumed that a package containing different components could be more effective than a single intervention to increase contraceptive uptake. Indeed, as sexual abstinence appeared to be supported by religious and traditional norms for up to three to 6 months in both study countries, the immediate postpartum period or the established six-week postpartum visit dedicated to contraceptive services may not respond to women’s demand. Instead of targeted “high-dose, low-frequency” strategies that promote a method over another and restrict services to a narrow window period, we adopted a “low-dose, high-frequency” approach to guide the selection of the interventions for the package, assuming it would be more effective. This approach is based on women’s choice and would be offered during the third trimester of pregnancy (to not burden already overstretched providers during first and second trimester visits) and the first year postpartum through iterative offering of contraceptive information and services. We also assumed that interventions that are chosen by and designed with key stakeholders will better reflect field reality and ensure its feasibility, sustainability, and scalability to other settings [29]. Therefore, we hypothesized that implementing an intervention package that is designed to strengthen health services during existing ANC and PNC visits, and that draws upon results of a formative study which accounted for the views of various stakeholders (including those of clients), is in line with national health policies, and takes into consideration the scarcity of public resources, would result in an increase in the uptake of contraceptive methods during the first year postpartum compared with the general standard of care. The package would comprise interventions that support service providers to carry out PPFP services according to national recommendations.

We recently published the research protocol of the YAM DAABO study [30], which was grounded in two conceptual frameworks: first, the WHO Health Systems Framework with its six system building blocks (leadership/governance, health care financing, health workforce, medical products and technologies, information and research, and service delivery) [31]; second, the WHO framework for ensuring human rights in the provision of contraceptive information and services [32]. The latter framework comprises the following elements: non-discrimination, availability, accessibility, acceptability, quality, informed-decision making, protection of privacy and confidentiality, participation, and accountability. Both frameworks are interrelated and informed the design of the PPFP intervention package and the research instruments. We aim to describe in this paper the detailed process of designing the PPFP intervention package in two countries and the content of each of its components.

### Study countries

The study takes place in Burkina Faso and the Democratic Republic of Congo (DRC), two countries with a high contraceptive unmet need among all married women of reproductive age (at 24 and 28%, respectively) and a critically high contraceptive unmet need among postpartum women (at 89 and 66% right after delivery, 50 and 48% after 6 months of lactational amenorrhea, and 21 and 36% at the end of lactational amenorrhea, respectively) [9, 33, 34]. A majority of women in Burkina Faso and the DRC attend ANC (95 and 88%, respectively) and deliver in a health facility (66 and 80%, respectively). In Burkina Faso, 72% of postpartum women receive PNC within 48 h of childbirth against 44% in the DRC, and 81% of children 12–23 months receive all recommended vaccines compared with 41% in the DRC.

## Methods

The design of our PPFP intervention package was based on participatory action research principles [35] and entailed two main strategies that were complementary: (1) a bottom-up formative approach applying qualitative research methods that engaged community members, service providers, and policymakers from Burkina Faso and the DRC to identify barriers and catalysts related to PPFP uptake; and (2) a circular reflective approach that involved health staff, members of the national research teams, and the WHO coordination team. Before the two main strategies unfolded, the research team also conducted in each country fact-finding field observation visits to three selected health centers to observe practices related to PPFP.

### Bottom-up formative approach

We started with formative activities to identify a package of PPFP interventions to be implemented in a cluster randomized controlled trial during the experimental phase. With a view to informing the design of the PPFP intervention package, we sought the participation of a variety of stakeholders to identify barriers and catalysts related to PPFP uptake. The formative activities took place in each country in three health centers and their respective coverage areas (these sites where different from those where field observation visits occurred). These centers were conveniently drawn from each country’s sampling frame which was established at the beginning of the study. The formative sites were in the province of Yako, Burkina Faso (communes of Bokin, rural; Latoden, rural; and Yako, urban) and in the city-province of Kinshasa, the DRC (districts of Nsele, rural; Kimbanseke, urban; and Bumbu, urban). We conducted in total 22 separate focus group discussions (FGDs) (*n* = 13 in Burkina Faso, *n* = 9 in DRC) of six to ten participants (total number of participants: *n* = 124 in Burkina Faso, *n* = 89 in DRC) for the following categories of individuals: (a) women who were currently pregnant or who delivered at the health center during the previous 12 months - their age determined the allocation to subgroups: adolescent girls (≤ 19 years), women 20–39 years, women ≥40 years (the chosen age limits were not dictated by obstetric risks but by the potential compatibility of women to participate and freely exchange in the same FGDs); (b) men (25–40 years) who were current partners or husbands of women who were pregnant or who delivered at the health center during the previous 12 months; and (c) service providers. We conducted 34 in-depth interviews (IDIs) (*n* = 21 in Burkina Faso, n = 13 in DRC) with clinic managers, national policymakers, and individuals whom local informants had identified in each recruitment zone as influential (such as religious leaders, village chiefs, community personalities, or others). Adult participants gave their written informed consent. Non-adult participants gave their written informed assent and their respective parents or guardians provided written informed consent.

The semi-structured FGD and IDI guides were prepared in French. In each country, interviews were conducted by a team of social scientists speaking the respective local languages (Mooré in Burkina Faso and Lingala in the DRC). The interviewers were trained to pay attention to finding the most appropriate and clear language to express technical terms in the respective local languages.

The interviews took place in a location guaranteeing privacy and were audiotaped after obtaining the agreement from participants. Research assistants transcribed and translated the audio records into French using Microsoft Word and ensuring that participants’ personal information was anonymized. Accuracy checks were done by comparing transcripts with audio files. We performed a thematic analysis using QSR NVivo 11 software, a qualitative research management tool. We established a basic codebook to describe all the nodes and used it to code data. The four main nodes were: (1) PPFP knowledge, attitudes, and practices; (2) perceived barriers and catalysts related to PPFP uptake; (3) strategic and programmatic considerations related to PPFP; and (4) quality of care pertaining to PPFP. We enriched the codebook with new emerging nodes during the coding process. Data were coded by research assistants using NVivo. To ensure quality of data coding, we first asked research assistants to code a common transcription sample and performed inter-rater reliability testing by computing the Cohen’s Kappa coefficient. We considered a Kappa coefficient ≥ 0.8 to be an acceptable concordance threshold.

The results of this bottom-up formative approach are summarized in Table 1. The Results section incorporates selected quotes that are relevant to the intervention design.

**Table 1:** Summary of perceived barriers and facilitators related to postpartum family planning (PPFP) in Burkina Faso and the DRC

Barriers • Lack of male engagement • Out-of-pocket co-payment of contraceptives • Women’s reliance on amenorrhea for pregnancy prevention without knowing its limits • Women’s and men’s misconceptions about modern contraception, including prerequisites for lactational amenorrhea method • Sexual abstinence supported by religious and traditional norms for up to three to six months (although women reported earlier resumption of sexual activities) • Low prioritization by women of scheduled postpartum visits • Limited availability of readily accessible methods and stock-outs in health facilities^a^ • Lack of dedicated PPFP counseling materials^a^ • Limited availability of clinic days and scheduled visits dedicated to contraception^a^	
Facilitators • Political will and enabling policies for family planning • Support from certain religious leaders and men • Negative traditional views on the consequences borne by closely-spaced children and their mothers • Six-week postpartum visit dedicated to contraception (albeit poorly attended)	

^**a**^Findings also identified during field observation visits in both countries

### Circular reflective approach

A research workshop gathered in Geneva, Switzerland, one service provider from a formative site in each country (we felt that service providers’ perspectives were sufficiently captured during qualitative interviews and that a provider by country could represent them during the workshop), five members from each country research team (including a clinical expert in contraception), and four members of the WHO coordination team. We estimated that the perspectives of women and other community members were largely represented during qualitative interviews. Therefore, we did not invite them to the research workshop.

We, researchers and workshop participants, took stock of the frameworks underpinning the overall study (see Background). We kept in mind that the proposed interventions should not add burden to the current organization of health services and to service providers. Interventions should help service providers to offer PPFP services which they are expected to offer as per national recommendations but cannot because of lack of human, material, or organizational resources. We reflected on the fact that there has been in recent years a renewed interest in highly effective long-acting and reversible methods, such as immediate postpartum IUD. Burkina Faso and the DRC recently gave commitments to pilot such programs as they showed promising results [22]. However, we reminded ourselves that this research adopted a “low-dose, high-frequencies” approach and that interventions to be identified should be low-cost, feasible in primary health centers, and strengthen health service delivery during client-provider encounters from the third trimester of pregnancy up to 12 months postpartum.

We presented the results of the FGDs and IDIs to allow everyone at the workshop – as individuals, members of country teams, and collectively – to reflect on their significance as possible interventions of the PPFP package. We were open to designing country-specific interventions based on key barriers and catalysts identified in each country. As results were far from being different between Burkina Faso and the DRC – and in fact from other similar Sub-Saharan settings, we contemplated the possibility of and agreed on crafting a common set of PPFP interventions to address the dominant gaps shared by both countries (health staff competencies in PPFP, availability of PPFP services, clients’ misconceptions about contraception, low prioritization by women of scheduled postpartum visits, and lack of male engagement). We thought that such package, if proven effective, could be more easily scaled up to other settings that share similar barriers and enablers. When examining the potential effectiveness of possible interventions, as well as acceptability to women and providers, feasibility, “integrability” into the RMNCH continuum, sustainability, and scalability, each country participant was iteratively invited to express his or her views on possible interventions, with the WHO team helping them to remain within the adopted frameworks and assumptions. To facilitate shared visualization and discussion, all proposed interventions were posted on a wall and prioritized according to the perceived level of potential impact, feasibility, and roughly-estimated high or low cost. Through repeated individual voting and negotiation processes to understand our differences in opinion and strengthen common grounds, we reached a consensus on which interventions could be discarded. We conducted this process several times to narrow down the number of proposed interventions before selecting the final set of PPFP interventions.

To gauge the operational feasibility and relevance of the components in the package, another reflective feedback loop occurred at country level involving the rest of the research teams and health staff, including clinic managers. Where appropriate, clients were mobilized to provide inputs, such as on the contents and comprehension of printed intervention materials.

## Results

Table 2 summarizes PPFP interventions that were debated and the main reasons why they were not retained (e.g., outside the scope of strengthening services within health centers, perceived as resource-intensive, or already studied).

**Table 2:** Summary list of initially-proposed postpartum family planning (PPFP) interventions in Burkina Faso and the DRC and main reasons why they were not retained

Initially-proposed PPFP interventions	Main reasons why they were not retained
Demand-side interventions	
Mass sensitization campaign (e.g., multimedia, community-based champions)	Outside the scope of the research project; perceived as resource-intensive; researched elsewhere [39, 40]
Community-based interventions for men, such as “école des maris” (school for husbands) or through religious or community leaders	Outside the scope of the research project; perceived as resource-intensive; researched elsewhere [41, 42]
Outreach interventions through community health workers	Outside the scope of the research project; perceived as resource-intensive; researched elsewhere [43]
Appointment reminders through mobile or fixed telephone calls or messaging	Time-consuming and costly for the clinic and not feasible due to the weak level of telephone ownership at the community level
Supply-side interventions	
Increasing the number of trained providers in health centers	Perceived as not sustainable
Integration of PPFP into pre-service training	Not feasible within the scope, timeframe, and funding of the research project
Integration with immunization programs	Perceived as not feasible and costly due to limited human and financial resources; researched elsewhere [44]
Free contraceptive methods	Perceived as not sustainable
Postpartum intra-uterine devices	Procedure not allowed to be performed by auxiliary midwives who are the main staff offering reproductive, maternal, newborn, and child health services at the primary healthcare level

The six interventions of the final package can be presented from the perspective of the supply side on the one hand (factors related to the provision of health services), and demand side on the other (factors related to the demand of health services by clients). The supply side comprises the following interventions: (1) PPFP refresher training of service providers, (2) regularly scheduled and strengthened supportive supervision of service providers, and (3) improved availability of PPFP services 7 days a week. The demand side comprises the following interventions: (1) appointment cards for women and (2) invitation letters for partners. The sixth intervention, a PPFP counseling tool, addresses both supply and demand sides.

### PPFP refresher training

#### Situation

Service providers working on RMNCH at the primary healthcare level in Burkina Faso and the DRC are mostly auxiliary midwives (“accoucheuses”) whose curriculum is shorter than the midwifery curriculum and allows them to perform normal deliveries and offer basic contraceptive methods, including injectables and implants (but usually not IUDs, which are offered by midwives or higher-level health cadres). Additional in-service training appeared to be necessary, as reported by a policymaker in the DRC:

*There are new graduates who are starting to work. Although they attended a professional school, further training is needed for them to acquire family planning competencies. The actual basic training is not adapted to the current needs. Therefore, there is always a need for additional training.*

Recently, Burkina Faso and the DRC have demonstrated the political will to strengthen PPFP competencies of service providers during in-service training. This will has not yet cascaded into action, and field observation from our research teams suggested that knowledge and skills in FP, and in particular PPFP, remained uneven among service providers working in primary healthcare settings.

#### Action

In light of the situation, the workshop participants agreed on the need to translate into action the national political will regarding PPFP, and refresh and enhance PPFP knowledge and competencies of service providers. As part of the intervention package and with the support of WHO, the co-principal investigators (co-PIs), who are both clinical FP experts, will design and implement a six-day interactive and hands-on curriculum targeting the providers of the health centers in the experimental group. The curriculum will be mainly centered on effective counseling and the use of the PPFP counseling tool (see below). Training sessions will include interactive presentations, role-plays with the PPFP counseling tool, and clinical practice.

### Supportive supervision

#### Situation

National policies in Burkina Faso and the DRC recommend regular supportive supervision visits. These visits are mostly used for data collection. Limited local resources and skills hamper the regularity of these visits as well as the integration of other tasks into its objectives, such as monitoring the quality of services, motivating staff, or problem-solving. Providers in the DRC reported this gap, as follows:

*Providers’ competencies are often not kept up to speed and providers are not supported or accompanied. If there is an organization that targets the health facility for a specific activity, then we get trained again. Apart from that, there is nothing – no staff development plan.*

#### Action

The workshop participants concurred with the fact that PPFP should be addressed during regular supportive supervision visits and should, therefore, be part of the PPFP package. This can be done with few additional resources: the co-PIs who are clinical experts in family planning and supervision will accompany the respective local RMNCH supervisors in their supportive supervision visits and will strengthen their supervisory skills related to FP in general, and to PPFP in particular. They will ensure that quality of care and staff motivation are addressed during each site visit. Health centers will benefit from the enhanced supportive supervision for a day every 3 months.

### Availability of PPFP services

#### Situation

Field observation from our research teams in Burkina Faso and the DRC indicated that contraceptive services were generally available only during certain days or half days of the week, similar to other Sub-Saharan countries. Contraceptive supplies are often locked away in another part of the health center and not available in the immediate postpartum, as reported by a provider in Burkina Faso who simply proposed “*to be allowed to stock [FP] supplies at the maternity.*”

#### Action

Contraceptive services will be made available at the health facility 7 days a week for all postpartum women. The workshop team considered that this intervention could be implemented with little additional resources: staff on duty at the maternity or for postpartum services will receive PPFP refresher training and supportive supervision in the context of the PPFP intervention package (see above); and contraceptive methods, which are stored (and often locked away) in health centers, can be easily made available 7 days a week with little extra effort in terms of logistics management.

### PPFP counseling tool

#### Situation

Based on field observation from our research teams, service providers did not appear to integrate systematically PPFP into ANC, postpartum care, or PNC, although PPFP information and services ought to be part of the RMNCH continuum of care according to national recommendations. We observed the presence but not necessarily the use of job aids that contained messaging on HIV, maternal and newborn health, or family planning in general. There was no dedicated PPFP counseling tool to help providers counsel women and address misconceptions specifically on this topic. Providers in the DRC expressed the gap as follows:

*We need counseling tools to explain clearly to women because some are slow to understand, and others think that these [contraceptive] methods can make them sterile. We can use images to give information on method use and how to remove it if the couple wants to conceive again.*

#### Action

In order to help providers do what they are expected to do, we designed and piloted a *PPFP counseling tool for service providers and their clients*. The tool will be used during the antenatal period (third-trimester visits), after childbirth and before discharge from the health facility, and during subsequent postpartum visits. We chose to use the tool only with women in their third trimester and onward for the following reasons: (1) mixed results of studies looking at the impact on postpartum contraceptive use of interventions starting before or during the third trimester [11–15]; (2) avoid burdening with PPFP counseling messages already overstretched service providers who see women in their first and second trimester. The development process and detailed contents of the tool was described in another article [36].

### Appointment cards

#### Situation

Postpartum women did not appear to prioritize the importance of recommended scheduled visits, as suggested by providers in Burkina Faso:

*For postpartum family planning visits, the proportion is very low. Women do not come during the postpartum. But if it’s a visit for the infant, then they all come…[Women] tell themselves that once they have given birth, it’s over and they have no reason to come…It’s the community health worker who does outreach visits who has to catch up with them at home.*

Our PPFP service model is based on national policies and spans over several visits covering the period from the third trimester of pregnancy up to a year after childbirth. As such, adherence to scheduled visits is important for women to receive PPFP information and services that are appropriate to their postpartum age and breastfeeding situation. There is in general no established reminder system for clinic appointment: service providers usually just tell women when to return for follow-up. As access to fixed telephone lines or personal mobile phones is limited, SMS or reminder calls are not feasible, in addition to being costly and time-consuming for clinic staff.

#### Action

The workshop participants agreed that giving simple appointment cards requires little additional resources and that these cards could be given to women at each follow-up visit to remind them of the following ones. The card would also contain a message to stress the health benefits of follow-up visits. After the workshop, both country teams proposed a few models. We eventually adopted two types of appointment cards: one for ANC and another one for PNC, both of which were tested with women and service providers for cultural appropriateness (see Additional file 1).

### Invitation letter for partners

#### Situation

The lack of men engagement and the absence of interventions that specifically target them were found in our formative research to be a key barrier to PPFP uptake. A woman from the DRC shared her fear during an FGD:

*Our husbands are the problem. You did not see how I hid when my husband just passed by (during the FGD). If he sees us, he will think that I came to get a birth spacing method, and tonight he would beat me.*

In contrast, a man in Burkina Faso said during an FGD:

*If they (the providers) invite us, we would be willing (to participate). But they invest more on women; for men, they do nothing for the moment, but men also want support.*

The workshop participants debated several possible interventions to engage men, such as “école des maris” (school for husbands) or other forms of discussion groups which engage men and are facilitated by community health workers. We concluded that these activities, while promising in terms of effectiveness, require significant resource investment. They also fall out of our research frame, which is to strengthen what is being done or should be done – in health facilities – to support access to PPFP information and services.

#### Action

In contrast, we concurred with the fact that counseling men alone or in couple at the health facility is feasible and affordable. In order for partners to benefit from messages contained in our PPFP counseling tool and targeting men and couples, they need to come to the health facility – alone or in couple. International experiences on the engagement of women’s partner in the prevention of mother-to-child transmission (PMTCT) of HIV or voluntary counseling and testing (VCT) for HIV testing during ANC showed that invitation cards for men are cost-effective [37, 38]. The workshop team felt that a similar intervention could have the potential of strengthening the participation of partners in PPFP. We agreed on an official letter of invitation and its contents, which were pilot-tested with men, women, and service providers for cultural appropriateness immediately following the workshop (see Additional file 2). Service providers will give this letter of invitation to women when they attend services in the third trimester of pregnancy. Based on WHO recommendations on involving men in the health promotion interventions for maternal and newborn health, we will ensure that the following precautions are taken when implementing this intervention, so that women’s confidentiality, security, privacy, and autonomy are respected: (1) women have the freedom to choose whether to give or not the invitation card to their partner; (2) couple counseling will be part of the refresher training for service providers; (3) health staff and research assistants based at the health center will be trained on the following points: (a) none of them have a role in contacting the partners, (b) women are free to make their own decision whether or not to involve their partners, (c) women are informed that the participation of their partners is not at all mandatory, (d) even if a partner has responded positively to the invitation letter at the beginning of the study, every time he accompanies the woman to attend the visit, service providers will first ask for the woman’s agreement before allowing the partner to attend the visit.

## Discussion

The unmet need for postpartum family planning remains unacceptably high, despite the proven benefits of modern contraceptive use. Our research strategy assumes that this unmet need can be addressed by reinforcing the existing service delivery model at the primary healthcare level with a series of low-cost and low-technology interventions chosen with the input of stakeholders. This would in turn help service providers apply national recommendations. Our approach does not promote a modern contraceptive method over another but takes into consideration the paramountcy of women’s right to information and choice regarding postpartum fertility options. This may constitute an appropriate pathway to respond to the wishes, decisions, and situations of each postpartum woman and couple by giving them information, including on the benefits and limitations of PPFP and of available contraceptive methods, and letting them choose. The study proposes to implement six interventions subsumed into a package that could contribute to reinforcing existing ANC and PNC services in primary health clinics and thereby increasing PPFP uptake. This approach departs from other earlier studies presented under the "Background" section that have focused upon a specific contraceptive method or a single programmatic strategy. In addition, with the view to designing contextually-sensitive interventions that are informed by human rights principles as outlined by WHO, we adopted an inclusive process and used two interlinked approaches to discuss options with country stakeholders before identifying the final interventions of the package. We applied participatory action research principles: a bottom-up approach, which engaged a wide range of stakeholders, including women, adolescent girls, men, other community members, service providers, policymakers; and a circular reflective approach with providers and researchers from countries and WHO. In this paper, we aimed to present this process and the detailed contents of the PPFP package.

### Limitations

In designing the package, we faced the possibility of developing multiple sets of interventions for different settings. Yet our formative research showed that challenges and opportunities related to PPFP uptake in Burkina Faso and the DRC were far from being dissimilar and generally reflected the situation in other parts of Sub-Saharan Africa. Despite shared barriers and catalysts in both countries, stakeholders could have had diverse views on the cost-effectiveness of different interventions to address these obstacles, which could have led to a differentiated choice of interventions for each setting. However, the primary healthcare context of the research, the focus on reinforcing existing ANC and PNC services in health centers, and the considerations related to feasibility, sustainability, cost, and scalability led us to discard interventions that did not fulfill these conditions, and limited our options to six low-cost, low-technology, and easily replicable interventions that addressed the main service delivery barriers related to PPFP found in both countries.

Women and community members were not part of the intervention design workshop, and only one provider per country attended it. However, we felt that qualitative interviews sufficiently captured the various perspectives of relevant stakeholders. Furthermore, another feedback loop at field level engaged women and other health staff to share their views on the operational feasibility and acceptability of proposed interventions.

The effectiveness of this PPFP intervention package remains to be tested in the experimental phase through a cluster randomized controlled trial. This trial is however not designed to determine which components of the intervention package will have an effect, or not, on contraceptive uptake. To this end, we have embedded qualitative research in the experimental phase as well as process-oriented indicators in the quantitative research instruments (case-report forms). This will allow us to assess which intervention in the package is suggested to have a greater effect, or not, on postpartum contraceptive uptake.

In terms of public health intervention, program managers and policymakers may prefer just a single one-size-fits-all cost-effective intervention to a package comprising different components. However, considering the social, cultural, and religious challenges and opportunities surrounding contraception, we believe that our operational research with its participatory approach and resulting low-cost, low-technology intervention package merits study as the interventions were designed to be easily replicable and scaled up in settings with similar characteristics.

### Implications

If found to be effective, this intervention package may be relevant to and scalable in other parts of Burkina Faso and the DRC. Before scaling up to other Sub-Saharan countries that have comparable profiles and are interested in adopting such an approach to address the unmet need for postpartum contraception, the package would require language and cultural contextualization to account for local specificities. Further implementation research may be required. In addition, proposed interventions that were not retained deserve to be reviewed and integrated into future PPFP research that focus on higher-resource settings.

## Conclusions

A participatory inquiry and reflective process engaging a wide range of stakeholders resulted in a package of interventions that aimed to strengthen ANC and PNC services at the primary healthcare level, and support providers in offering PPFP information and services. The intervention design was aligned with national policies and underpinned by considerations related to scarcity of resources, feasibility, sustainability, and scalability to other settings, whilst being informed by human rights principles. The impact of the package on postpartum contraceptive uptake will be studied in a cluster randomized controlled trial.

## Additional files

Additional file 1: Appointment card for women. (PPTX 100 kb)

Additional file 2: Invitation letter for partners. (DOCX 34 kb)

## Abbreviations

ANC: Antenatal care
DRC: Democratic Republic of Congo
FGD: Focus group discussion
FP: Family planning
IDI: In-depth interview
IUD: Intra-uterine device
LAM: Lactational amenorrhea method
PNC: Postnatal care
PPFP: Postpartum family planning
RMNCH: Reproductive, maternal, newborn, and child health
