# Supplementary material for: Participatory action research to identify a package of interventions to promote postpartum family planning in Burkina Faso and the Democratic Republic of Congo
Source: BMC Womens Health. 2018 Jul 5;18:122. doi: 10.1186/s12905-018-0573-5 (PMC6034289; doi:10.1186/s12905-018-0573-5)
Supplement: Supplementary file 1 — Appointment card for women. (PPTX 100 kb) [file 12905_2018_573_MOESM1_ESM.pptx]

## Slide 1
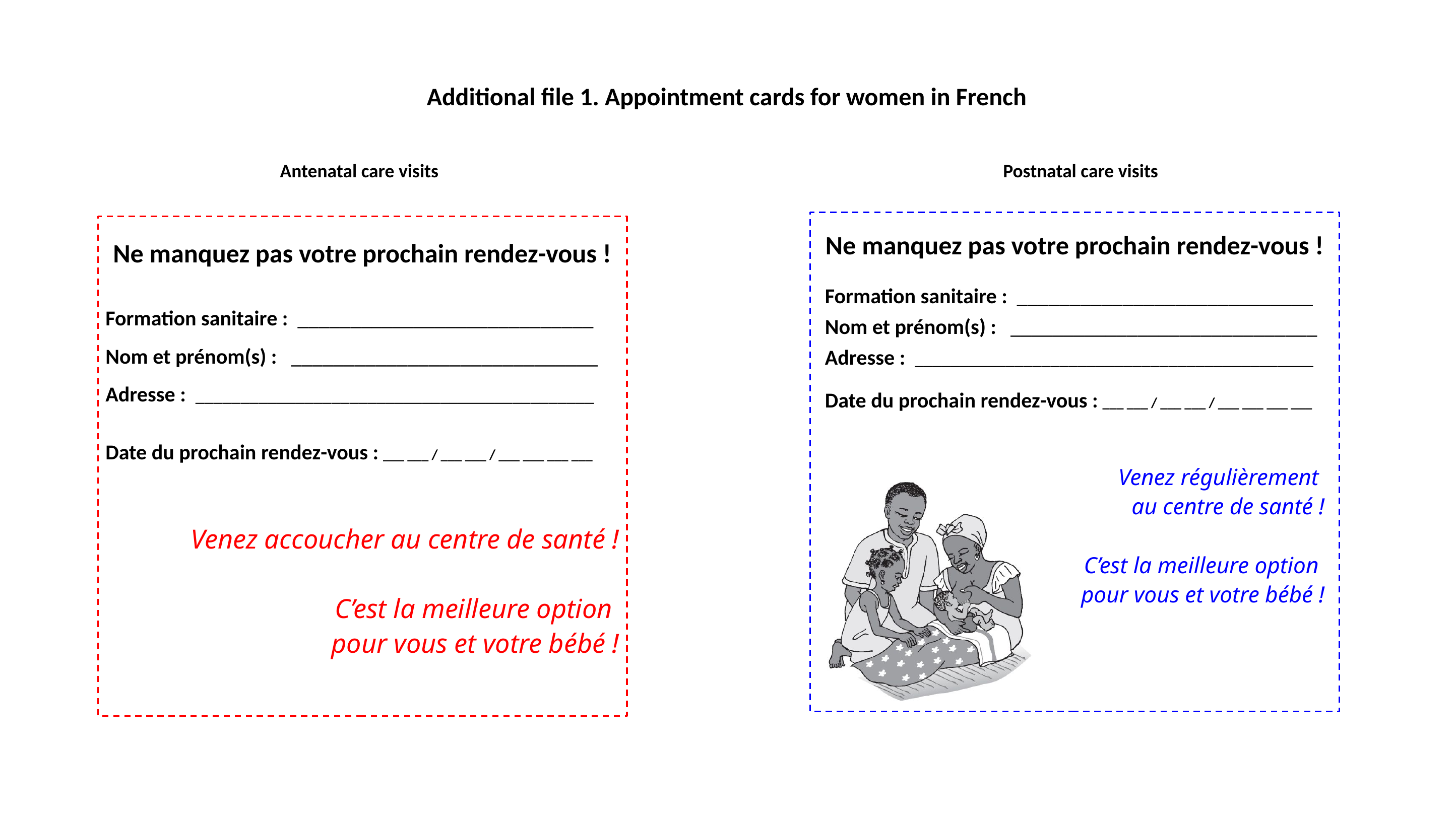

Additional file 1. Appointment cards for women in French
Antenatal care visits
Postnatal care visits
Ne manquez pas votre prochain rendez-vous !
Formation sanitaire : ____________________________
Nom et prénom(s) : _____________________________
Adresse : ____________________________________________
Date du prochain rendez-vous : ___ ___ / ___ ___ / ___ ___ ___ ___
Venez régulièrement
au centre de santé !
C’est la meilleure option
pour vous et votre bébé !
Ne manquez pas votre prochain rendez-vous !
Formation sanitaire : ____________________________
Nom et prénom(s) : _____________________________
Adresse : ____________________________________________
Date du prochain rendez-vous : ___ ___ / ___ ___ / ___ ___ ___ ___
Venez accoucher au centre de santé !
C’est la meilleure option
pour vous et votre bébé !

## Slide 2
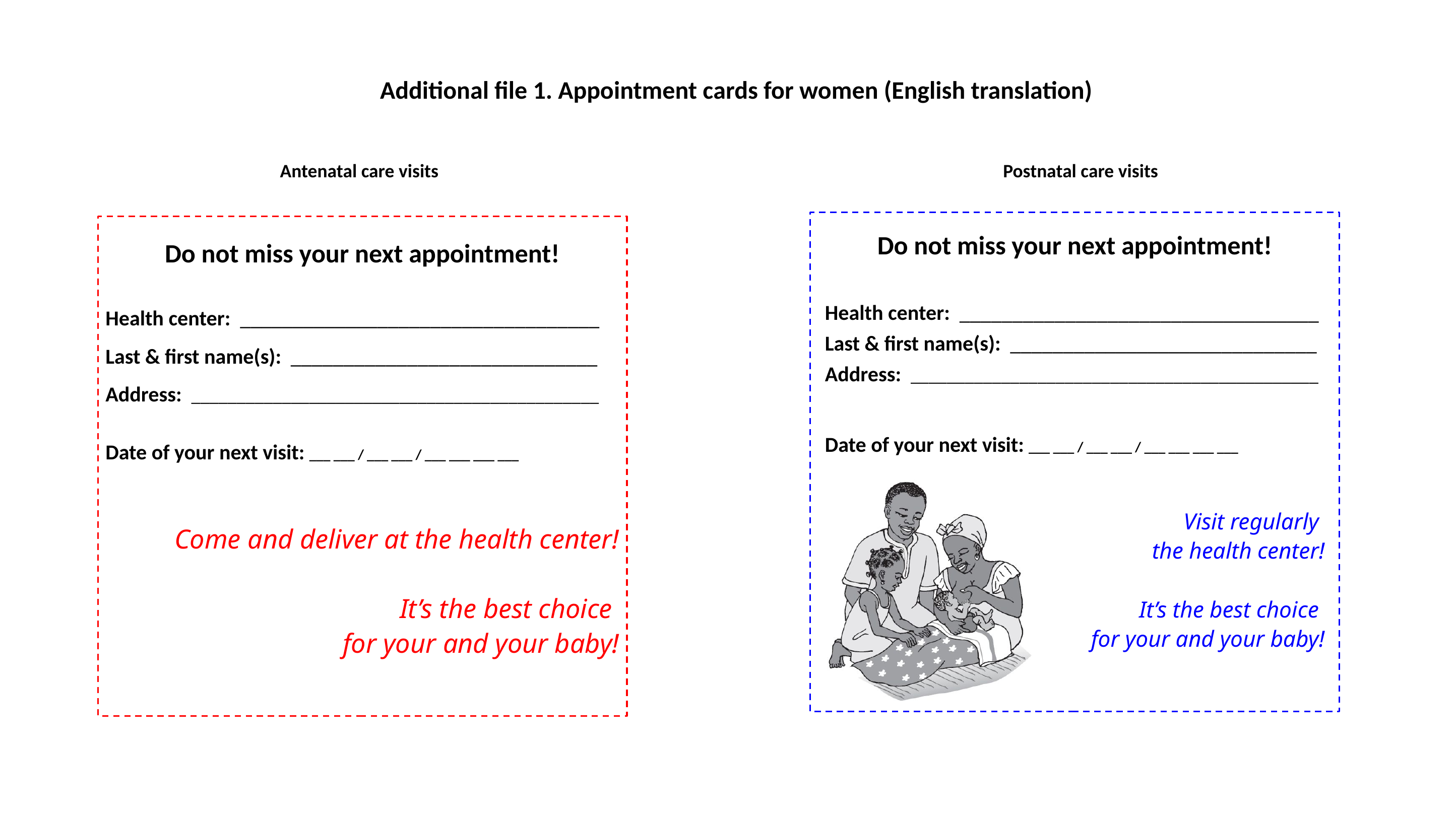

Additional file 1. Appointment cards for women (English translation)
Antenatal care visits
Postnatal care visits
Do not miss your next appointment!
Health center: __________________________________
Last & first name(s): _____________________________
Address: _____________________________________________
Date of your next visit: ___ ___ / ___ ___ / ___ ___ ___ ___
Visit regularly
the health center!
It’s the best choice
for your and your baby!
Do not miss your next appointment!
Health center: __________________________________
Last & first name(s): _____________________________
Address: _____________________________________________
Date of your next visit: ___ ___ / ___ ___ / ___ ___ ___ ___
Come and deliver at the health center!
It’s the best choice
for your and your baby!
