# Supplementary material for: Participatory action research to identify a package of interventions to promote postpartum family planning in Burkina Faso and the Democratic Republic of Congo
Source: BMC Womens Health. 2018 Jul 5;18:122. doi: 10.1186/s12905-018-0573-5 (PMC6034289; doi:10.1186/s12905-018-0573-5)
Supplement: Supplementary file 2 — Invitation letter for partners. (DOCX 34 kb) [file 12905_2018_573_MOESM2_ESM.docx]

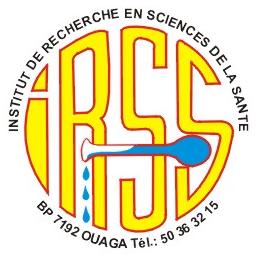
***Lettre d’invitation***

**District sanitaire de Yako**

**Centre de santé de : _____________________**

Cher Monsieur __________________________________________,

La grossesse de votre épouse, Mme _________________________ , tend vers sa fin. Cette grossesse a évolué normalement. L’accouchement se fera bientôt et nous aimerions vous aider à le préparer au mieux pour assurer le bien-être de votre épouse et de votre enfant.

C’est pourquoi, nous vous invitons à accompagner votre épouse au centre de santé lors de sa prochaine visite fixée pour le :

Date : ___________________

Pour cet entretien, votre présence est importante et souhaitée.

En cas d’indisponibilité à la date indiquée, vous pourrez toujours accompagner votre épouse lors de ses prochaines visites au centre de santé.

Prière de nous ramener cette note d’invitation le jour du rendez-vous.

____________________________

Signature

**Additional file 2. Invitation letter for partners**


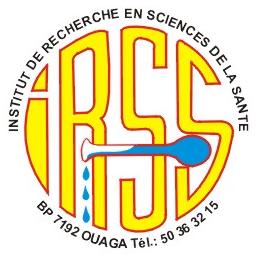
*[English translation]* ***Invitation letter***

**Yako Health District**

**Health center : _____________________**

Dear Mr. __________________________________________,

The pregnancy of your spouse, Mrs. _________________________, is coming soon to its term. Her pregnancy has progressed without problem and she will soon give birth. We would like to help you best prepare the birth to ensure the well-being of your wife and baby.

For this reason we would like to invite you to accompany your wife to the health center for her next visit on the following date:

Date: ___________________

For this visit, your presence is important and desired.

In case you are not free for this date, you can always accompany your spouse during her next visits to the health center.

Please bring back this invitation note on the day of the visit.

____________________________

Signature
